# Hand-Fixing

**Published:** 1905-04

**Authors:** 


### EDITORIAL NOTES AND COMMENTS.

The Business office of THE JOURNAL-RECORD is No. 1007-1008 Century Building.

The Editorial office is 318-319-320 The Prudential.

Address all Business communications to Dr. M. B. Hutchins, Mgr.

Make remittances payable to THE ATLANTA JOURNAL-RECORD OF MEDICINE.

On matters pertaining to the Editorial and Original Communications address Dr. Bernard Wolff, Atlanta.

Reprints of original articles will be furnished at cost price. Requests for the same should always be made *on the manuscript.*

We will present, post-paid, on request, to each contributor of an original article, twenty (20) marked copies of THE JOURNAL-RECORD containing such article.

### "HAND-FIXING."

WE are going to treat a new bunch of quacks to a little free advertising. If this notice should do them any good, we apologize—it was unintentional. The newest claimant to quack notoriety is one D. D. Palmer, who sets forth his titles and his "system" in the advertising columns of the *Medical Brief.* His system is termed *chiropractic*, which we are told means hand-fixing—not the removal of hangnails, or subungual alluvial deposits, but the replacing, fixing, adjusting, repointing, repairing (not to mention regretting—on the part of the patients) of luxated bones which have so pinched the "nerves of innervation," or "innate" nerves as to cause the greatest possible disturbance in that synthetic conception somewhat airily termed "the system."

Palmer is in position to speak authoritatively in this matter, for he is the original chiropractor, in fact, the founder of the school of chiropractic, which at present has its headquarters (and probably its fore and hind quarters also) at Davenport, Ia., U. S. A. He insists upon a careful distinction between chiropractic and osteopathy, drawing also the allopaths, as he is pleased to designate them, into this strange company for contrast. His system does not treat effects, diseases, people or anything else. Effects alone can be treated. Causes can not be treated. He is concerned only with causes and as they can not be treated, he treats nothing. It is something like that. We can not pretend to penetrate the subtlety of his reasoning. Chiropractors find " that all illness, sickness, affections, indisposition, complaint, maladies, or lack of innervation are but the results or consequences of disorder, derangement of some part of the human frame." If the human frame comprises the draping and the stuffing thereof, we are not disposed to controvert this very liberal view of things.

But the chiropractor forsakes the sure ground of safe generalities when he comes down to details. In his description of diphtheria there are quite a number of little things that arrest the attention as being somewhat at variance with modern if allopathic views. His description, despite his emphatic claim of particularity and separateness, borders very near the osteopathic notion of this disease. As they are both destined to be poured down the same sewer-hole, it is perhaps superfluous to raise an issue on this. Nevertheless, he says in speaking of the bacilli, which are reputed in non-chiropractic circles to be the cause of diphtheria, that they are the result of dead matter, like the "mold found on decaying cheese." Chiropractors understand that the necrosed membrane is a result of excessive heat, commonly named inflammation; that diphtheria is caused by those nerves which end in the membrane of the throat being pressed upon in a right dorsal foramen (as there are an even dozen of these foramina, it would seem important to learn which one was responsible for diphtheria) "which is occluded because of deleterious substances acting upon the sensory nerves, which in turn attack the motor. Abnormal sensations produce abnormal actions. This abnormal sensation and action acts on the adjacent vertebræ, displacing them so as to rick the nerves, which express

3

their injury by their twig ends being inflamed. The chiropractor replaces the displaced vertebra by one move which is distinctly chiropractic and cures diphtheria in one or two days."

Palmer rests his case with the concluding query, which rings with the note of challenge: "Wherein does the Chiropractic resemble the Allopath or the Osteopath?" One can not answer for the chiropractic and the osteopath—they will probably run together like two beads of quick-silver to form some sort of hermaphroditic union; but if the allopath were forced to reply categorically to this question, he would say that chiropractic bore about the same resemblance to allopathy that the alvine dejection does to the individual from whom it is discharged. The great marvel of all this is not that the *Medical Brief* should lend its aid to the exploitation of such charlatanry, but, in this day of enlightenment, cheap books, cheap newspapers, easy avenues for dissemination of knowledge, that any one should be so stupid as to be taken in by such a ghastly outfit as chiropractic offers.

But there is no doubt of the existence of such people, and in number sufficient to enable the chiropractor ere long to seek legal status through State legislatures, and have his board of examiners established to pass judgment on the neophyte's ability to locate the "nerves of innervation" or "innate nerves," and to answer the delicate question *which* dorsal foramen is responsible for diphtheria.

The hope is that with this and its like, a united medical profession will deal with it as Samuel dealt with Agag, and there shall be no more impatient waiting until truth-disclosing time shall expose such heresies, and in doing so annihilate them.

---

THE Georgia State Commission on Tuberculosis will meet in the Senate Chamber of the House of Representatives on April 18, 1905, at 3 p. m. Important business is before the commission, and a full attendance is desired.

